# *EGFR*双突变非小细胞肺癌患者临床特征分析

**DOI:** 10.3779/j.issn.1009-3419.2018.08.05

**Published:** 2018-08-20

**Authors:** 梦瑶 王, 敦强 任, 彩宏 郭, 晓倩 丁, 红梅 王

**Affiliations:** 266003 青岛，青岛大学附属医院呼吸科 Department of Respiration, Affiliated Hospital of Qingdao University, Qingdao 266003, China

**Keywords:** 肺肿瘤, EGFR, 双突变, 靶向治疗, Lung neoplasms, EGFR, Double mutation, Target therapy

## Abstract

**背景与目的:**

非小细胞肺癌（non-small cell lung cancer, NSCLC）中表皮生长因子受体（epidermal growth factor receptor, *EGFR*）常见单突变患者临床特征研究已经得到广泛认可，女性、无或少吸烟史、腺癌患者中发生率高，经EGFR酪氨酸激酶抑制剂（tyrosine kinase inhibitors, TKIs）治疗后客观缓解率（objective response rate, ORR）和无进展生存期（progression-free survival, PFS）显著延长。但针对*EGFR*双突变患者的临床特征以及对EGFR-TKIs治疗的敏感性研究尚不明确。本研究探讨了*EGFR*双突变患者的临床特征及EGFR-TKIs治疗的疗效。

**方法:**

收集2015年1月1日-2016年12月31日就诊于青岛大学附属医院接受*EGFR*基因检测的1, 238例原发性NSCLC患者，存在单一突变患者603例，双突变患者59例。所有基因均统一采用ARMS-PCR技术检测。对已明确具体基因分型的32例双突变患者进行基因分析，并随机选取60例单突变患者与59例双突变患者临床特征进行对照，同时比较晚期*EGFR*双突变和单突变患者对EGFR-TKIs治疗的疗效。

**结果:**

*EGFR*双突变患者中以合并罕见单突变基因最为常见（30/32）。双突变与单突变患者在性别、吸烟史、年龄、病理类型、肿瘤-淋巴结-转移（tumor-node-metastasis, TNM）分期等方面无显著性差异。在接受EGFR-TKIs治疗的双突变患者中，ORR为36.80%，疾病控制率（disease control rate, DCR）为68.40%，中位PFS为6.0个月，单突变患者中ORR为60.00%，DCR为90.00%，中位PFS为12.0个月，两组PFS比较有显著性差异（*P*=0.003）。

**结论:**

*EGFR*双突变患者的临床特征与单突变患者之间无显著性差异，双突变患者接受一代EGFR-TKIs治疗的ORR、DCR、PFS均较单突变患者低。

## Abstract (en)

**Background and objective:**

The clinical features of patients with common single-mutation of epidermal growth factor receptor (*EGFR*) in non-small cell lung cancer (NSCLC) has been well characterized. There is a high adenocarcinoma incidence rate among female patients with none or shorter smoking history. Those patients have higher objective response rate (ORR) and progression free survival (PFS) treated with EGFR tyrosine kinase inhibitors (EGFR-TKIs). However, it is still unclear that the clinical features of patients with *EGFR* double mutation and the sensitivity towards EGFR-TKIs treatment.

**Methods:**

We performed a retrospective cohort study of 1, 238 primary NSCLC patients who had *EGFR* gene testing in Affiliated Hospital of Qingdao University from January 1, 2015 to December 31, 2016 and identified 603 patients with single mutation and 59 patients with double mutation. All genes were uniformly detected by using ARMS-PCR technology. We analyze the gene of 32 double-mutant patients with specific genotyping, and randomly selected 60 patients with single mutation and compared the clinical features with 59 patients with double mutation. Furthermore, we examined the efficacy of EGFR-TKIs treatment in lung cancer patients with double mutation and single mutation in *EGFR*.

**Results:**

The rare single mutation gene is the most common in patients with double mutation of *EGFR*. There is no significant statistical difference in gender, smoking history, age, pathological type or tumor-node-metastasis (TNM) staging among patients with single and double *EGFR* mutantion. In the double mutation patients treated with EGFR-TKIs, the objective response rate was 36.80%, the disease control rate was 68.40%. The objective response rate was 60.00% and the disease control rate was 90.00% in the patients with single mutation. However, overall PFS was significantly higher in *EGFR* single mutation patients (*P*=0.003), with median PFS of 12.0 months compared with 6.0 months in *EGFR* double mutation patients.

**Conclusion:**

There was no significant difference between the clinical features of patients with *EGFR* double mutation and single mutation. Patients with *EGFR* double mutation is associated with poor survival underwent the first generation of EGFR-TKIs treatment compared with patients with a single mutation.

目前，肺癌是中国和世界范围内发病率和死亡率最高的肿瘤，多数确诊时已处于肺癌晚期，传统的放化疗疗效有限，中位生存时间仅为8个月-10个月，而随着人们对分子生物学及基因水平的研究，分子靶向治疗得到快速发展，使肺癌患者的无进展生存期（progression-free survival, PFS）及总生存期（overall survival, OS）延长，生活质量也有所提升。目前表皮生长因子受体（epidermal growth factor receptor, EGFR）是迄今为止非小细胞肺癌（non-small cell lung cancer, NSCLC）靶向治疗研究中最成功的受体之一。研究^[1]^发现亚洲人*EGFR*突变发生率达50%-60%，*EGFR*突变主要存在于18-21外显子中，其中19和21单一外显子突变最常见，约占*EGFR*总突变的90%。目前对于NSCLC中*EGFR*常见突变的研究已经非常广泛，大量文献报道得出*EGFR*常见突变在女性、无或少吸烟史、腺癌患者中发生率较高^[2]^。大量Ⅲ期随机临床研究已经证实：*EGFR*突变的NSCLC患者应用EGFR酪氨酸酶抑制剂（EGFR-tyrosine kinase inhibitors, EGFR-TKIs）治疗，PFS达到9个月-13个月，OS延长至20个月-30个月。基于这些临床研究已经将EGFR-TKIs纳入突变型晚期NSCLC一线标准化治疗^[3, 4]^。但随着研究的深入，越来越多罕见突变被发现，例如：18外显子的G719X、20外显子的S768I、21外显子的L861Q，还有我们目前已知的20外显子耐药基因T790M。除罕见单基因突变外，*EGFR*双基因突变也有相应文献报道过，但由于双突变的发生率低，样本量小，*EGFR*双突变患者的临床特征以及对EGFR-TKIs治疗的敏感性仍未完全明确。故本研究拟分析就诊于本医疗中心的59例*EGFR*双突变NSCLC患者的临床特征及EGFR-TKIs治疗疗效，从而为*EGFR*双突变患者提供临床数据及治疗依据。

## 1 资料与方法

### 1.1 研究对象

收集2015年1月1日-2016年12月31日就诊于青岛大学附属医院接受*EGFR*基因检测的原发性NSCLC患者，所有患者均经病理组织活检证实为NSCLC，且均由上海泛亚公司统一采用ARMS-PCR技术进行*EGFR*基因检测。对*EGFR*双突变和单突变患者的临床资料进行回顾性分析，比较*EGFR*双突变与单突变患者在临床特征上是否具有明显差异。根据实体瘤的疗效评价标准（Response Evaluation Criteria in Solid Tumor, RECIST）来评价使用EGFR-TKIs治疗的*EGFR*突变型患者的客观缓解率（objective response rate, ORR）、疾病控制率（disease control rate, DCR）、无进展生存期（progression-free survival, PFS），比较*EGFR*单、双突变患者治疗疗效之间的差异。

临床资料入组标准：具有完整的医疗资料，包括性别、年龄、吸烟史、病理类型、肿瘤-淋巴结-转移（tumor-node-metastasis, TNM）分期（TNM分期根据美国癌症联合委员会第七版TNM分类决定，以Ⅲa期为界，具体分为早期和晚期）。

治疗疗效入组标准：①确诊后一线经EGFR-TKIs治疗，每天服用吉非替尼250 mg *qd*、厄洛替尼150 mg *qd*或埃克替尼125 mg *tid*治疗，靶向治疗期间未接受其他放化疗、粒子植入及手术治疗。②具有规律的复诊记录，能提供相关检测数据（肺、颅脑、全腹、骨扫描等影像学及肿瘤标志物血液指标）。

### 1.2 统计学方法

数据应用SPSS 22.0软件进行分析，*EGFR*单、双突变与临床特征之间的相关性分析用卡方检验，*P* < 0.05为差异有统计学意义。*EGFR*单、双突变PFS之间用*Kaplan-Meier*法进行生存分析。

## 2 结果

### 2.1 *EGFR*突变频率

2015年1月1日-2016年12月31日就诊于青岛大学附属医院接受*EGFR*基因检测的原发性NSCLC患者共搜集到1, 238例，其中*EGFR*单突变患者603例，突变概率为48.71%；*EGFR*双突变患者59例（手术患者24例，非手术患者35例），突变概率为4.77%；双突变占*EGFR*总突变患者概率为8.91%；双突变患者中18/20外显子突变人数21例（35.59%），19/20外显子突变人数8例（13.56%），19/21外显子突变人数11例（18.64%），20/21外显子突变人数19例（32.20%）；由于临床资料在收集过程中存在部分数据丢失，59例双突变患者中明确具体基因分型的只有32例，32例双突变患者具体突变基因型分布见表 1。

**1:** 32例双突变患者具体基因型分布 Specific genotype distribution in 32 patients with double mutation

*EGFR* mutation	Mutation exon	Number	Percentage (%)
19del/L858R	19/21	2	6.3
19del/S768I	19/20	1	3.1
19del/T790M	19/20	4	12.5
G719X/S768I	18/20	14	43.8
S768I/L861Q	20/21	1	3.1
20-ins/L858R	20/21	1	3.1
S768I/L858R	20/21	1	3.1
T790M/L858R	20/21	8	25.0
EGFR: epidermal growth factor receptor.			

### 2.2 临床特征分析

59例双突变患者中有4例门诊失访，缺失完整的医疗资料，纳入双突变临床资料组患者共55例。从单突变患者中进行分层随机抽样，选取60例纳入单突变临床资料组。对两组患者的临床特征进行分析，包括性别、年龄、吸烟史、病理类型、TNM分期，基线特征见表 2。

**1:**
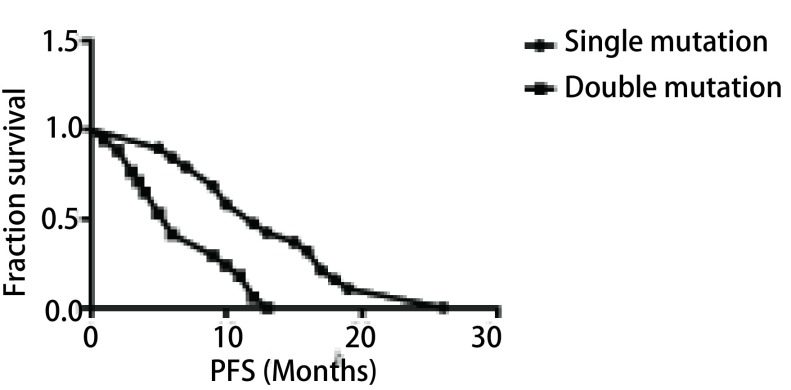
双突变组和单突变组无进展生存期比较 The comparison of progression-free survival in double mutation and single mutation

**2:** *EGFR*单突变和双突变患者临床特征比较 Comparison of clinical features of *EGFR* single mutation and double mutation

Characteristics	Double mutation (*n*=55)	Single mutation (*n*=60)	*χ*^2^	*P*
Gender			1.327	0.249
Male	26 (47.27%)	22 (36.67%)		
Female	29 (52.73%)	38 (63.33%)		
Smoking status			0.537	0.464
Smoker	21 (38.18%)	19 (31.67%)		
Non-smoker	34 (61.82%)	41 (68.33%)		
Age (yr)			0.773	0.379
> 60	32 (58.18%)	30 (50.00%)		
≤60	23 (41.82%)	30 (50.00%)		
Histology			1.866	0.497
Adenocarcinoma	55 (100.00%)	58 (96.67%)		
Squamous carcinoma	0 (0)	2 (3.33%)		
TNM staging			2.934	0.087
Early stage	26 (47.27%)	19 (31.67%)		
Later period	29 (52.73%)	41 (68.33%)		
TNM: tumor-node-metastasis.				

其中单突变资料组女性（63.33%）、无吸烟史（68.33%）、腺癌（96.67%）患者多见，18外显子突变1例（1.67%）、19外显子突变31例（51.67%）、20外显子突变2例（3.33%）、21外显子突变26例（43.33%），均与文献报道一致。双突变与单突变患者在性别、吸烟史、年龄、病理类型、TNM分期等差异均无统计学意义（*P* > 0.05）。

### 2.3 治疗疗效分析

双突变患者中共24例接受EGFR-TKIs治疗，包括非手术患者19例，术后复发患者5例。术后复发患者中1例靶向治疗同时联合放化疗，另1例同时联合粒子植入治疗。将仅接受EGFR-TKIs治疗的19例非手术患者纳入双突变治疗疗效组，从单突变患者中选取仅接受EGFR-TKIs治疗的20例非手术患者纳入单突变治疗疗效组，根据RECIST标准，回顾性分析两组的ORR、DCR及PFS。

#### 2.3.1 ORR、DCR比较

20例*EGFR*单突变治疗疗效组患者中，12例部分缓解（partial response, PR），6例病情稳定（stable disease, SD），2例病情进展（progressive disease, PD）。19例*EGFR*双突变治疗疗效组患者中，7例获得PR，6例SD，6例PD。将*EGFR*单突变组和双突变组进行比较，结果显示：ORR为60.00% *vs* 36.80%（χ^2^=2.092, *P*=0.205）；DCR为90.00% *vs* 68.40%（χ^2^=2.783, *P*=0.127）。差异均不具有统计学意义。

#### 2.3.2 PFS比较

双突变治疗疗效组平均PFS为7.4个月，中位PFS为6.0个月，单突变治疗疗效组平均PFS为13.9个月，中位PFS为12.0个月，两组比较有显著差异（χ^2^=8.863, *P*=0.003）。术后复发仅接受EGFR-TKIs治疗的3例双突变患者中，平均PFS为17.0个月，中位PFS为15.0个月。具体包括3种不同基因分型，突变基因为S768I/L861Q型患者，接受克唑替尼治疗，PFS为15个月，突变基因为G719X/S768I型患者，接受厄洛替尼治疗，PFS为26个月，突变基因为19del/L858R型患者，接受克唑替尼治疗，PFS为10个月。

## 3 讨论

本研究单突变临床资料组符合女性、无吸烟史、腺癌患者发生率高，19和21外显子位点突变共占95%，这与目前众多文献得出的结论一致。双突变临床资料组与单突变临床资料组进行比较，*P*值均大于0.05，说明双突变患者在性别、年龄、吸烟史、病理类型、TNM分期上与单突变患者不具有显著性差异。周欣^[5]^曾在文章中报道过6例L858R复合双突变患者与18例单突变患者的临床特征，发现单双突变之间无显著性差异，这与本研究结果相一致。

本研究应用EGFR-TKIs治疗的单、双突变患者中，单突变和双突变患者的ORR（60.00% *vs* 36.80%），DCR（90.00% *vs* 68.40%），可能由于本研究样本量较小，两组比较差异无统计学意义（*P* > 0.05），但*EGFR*双突变患者应用EGFR-TKIs治疗的有效率明显低于单突变患者。单突变患者的中位PFS为12.0个月，双突变患者中位PFS仅为6.0个月，与单突变患者比较（*P* < 0.01）具有显著差异。提示*EGFR*双突变晚期NSCLC患者应用EGFR-TKIs治疗的预后不如*EGFR*单突变患者。本研究收集18/20、19/20、19/21、20/21外显子双突变患者例数分别为21例、8例、11例、19例，而相同突变型之间的PFS具有一定的差异性，可能与具体基因分型有关。双突变的具体基因型是多样的，可为常见单突变复合常见单突变、常见单突变复合罕见单突变、罕见单突变复合罕见单突变，也可能是合并已知耐药基因的复合突变。本研究中已知具体基因分型的32例双突变患者中只有2例为常见单突变基因复合（19del/L858R），其他均复合罕见单突变基因，主要包括G719X、S768I、T790M、20-ins、L861Q，其中S768I/G719X双突变类型最常见，达到43.8%，这与Chen^[6]^的部分研究相吻合，他认为发生双突变的基因多复合罕见单突变基因，罕见单突变基因相较于常见突变基因，致癌基因较弱，往往不能单独导致肿瘤发生，需要复合成为双突变基因才能导致肿瘤的形成。杨雪等^[7]^也曾得出双突变中以S768I合并G719X最为常见的结论。本研究中单突变和双突变治疗疗效组之间存在显著性差异，可能与双突变组易复合罕见突变基因有关。常见突变基因如19位点的缺失突变和21位点的L858R点突变经EGFR-TKIs治疗疗效已经得到充分证实，能使PFS和OS时间明显延长，但目前对于罕见型*EGFR*突变经EGFR-TKIs治疗预后都较差。此前大量研究认为20外显子的插入突变和20外显子的T790M突变对EGFR-TKIs耐药^[8, 9]^。T790M突变的作用已被证实与原发性和获得性耐药相关，应用EGFR-TKIs治疗6个月-10个月后可出现T790M获得性耐药突变。对于20外显子插入突变，Woo等^[10]^报道过6例，应用EGFR-TKIs治疗整体预后都较差，其中有2例在治疗1个月内即出现疾病进展。Wu^[11]^报道过2例，应用一代EGFR-KTIs治疗后的PFS分别为1个月和1.5个月。由此可看出20外显子插入突变患者EGFR-TKIs治疗效果差。日本的Staoshi^[12]^在NEJ002研究中，发现罕见突变G719X或者L861Q突变的患者应用吉非替尼治疗后，中位PFS为2.2个月，中位生存期为12个月，总生存期明显短于常见突变，并认为具有少见突变的NSCLC患者接受一线化疗比接受一线靶向治疗效果更好。韩国的Baek等^[13]^研究的13例具有G719X或者L861Q的单一突变或者复合突变的患者，应用EGFR-TKIs治疗后，中位PFS为5.1个月，中位生存期为18.6个月。Kancha等^[14]^通过比较EGFR单突变对不同EGFR-TKIs的50%抑制浓度（half maximal inhibitory concentration, IC_50_），得出L861Q对于一代靶向药物均不敏感，需要较高的药物浓度来达到和常见突变一样的抗肿瘤效果，而S768I和L861Q相比较，IC_50_则更高，表明S768I可能对一代靶向药物耐药。段桦^[15]^通过分析国内外文献得出S768I单一突变患者较常见突变对一代EGFR-TKIs应答差。通过以上众多研究我们可以得出*EGFR*罕见突变对EGFR-TKIs治疗效果的敏感性不如常见突变。本研究中19例非手术双突变患者一线均接受一代EGFR-TKIs药物治疗，中位PFS仅为6.0个月，推测可能与罕见单突变基因影响了常见突变基因对EGFR-TKIs敏感性有关，具体机制有待进一步研究。

目前随着分子水平研究的深入，大量研究已经提出肿瘤异质性与治疗疗效以及耐药性相关，Gerlinger等^[16]^对4例肾癌患者的30个肿瘤样本进行分析，发现26个样本具有不同的等位基因，甚至在同一肿瘤的不同部位中，检测出预后良好和预后不良的基因。本研究中*EGFR*基因检测均来自于组织活检，采用ARMS-PCR技术，由于肿瘤异质性，利用小块组织检测到的变异可能无法完全反映病灶部位全部细胞变异情况，检测到的*EGFR*突变丰度也会具有一定的异质性。目前众多关于*EGFR*突变丰度的研究认为，*EGFR*突变基线丰度值可能与EGFR-TKIs治疗疗效呈正相关，*EGFR*突变丰度变化还可以反映疾病动态变化。Yang^[17]^的研究发现高丰度突变患者比低丰度突变患者具有更好的PFS，在经EGFR-TKIs治疗后逐渐进展的患者中，突变丰度降低的患者组获得更好的PFS和OS。由此我们可以推断本研究中相同基因分型患者之间PFS存在差异的原因可能与肿瘤的异质性有关。

目前相关研究已经得出*EGFR*罕见突变患者应用一代EGFR-TKIs治疗，敏感性介于敏感突变和野生型之间，而二代EGFR-TKIs治疗可以获得更好的预后。Yang等^[18]^证实了二代EGFR-TKIs阿法替尼对于G719X罕见突变型的客观缓解率达78%，中位PFS可达13.8个月，明显优于一代EGFR-TKIs。Kancha等^[14]^通过试验也证实了罕见突变接受一代EGFR-TKIs治疗有效率低。杨雪^[7]^分析了不同类型的*EGFR*少见突变基因，发现大部分携带少见突变的患者接受EGFR-TKIs治疗的ORR和PFS均较经典敏感性突变低，但较EGFR野生型患者高，与一代EGFR-TKIs相比，二代EGFR-TKIs可能更适用于*EGFR*少见突变的治疗。本研究中双突变患者一线均采用一代EGFR-TKIs治疗，无论是ORR、DCR还是PFS，均较*EGFR*单突变患者低。其中2例进展后更换为二代EGFR-TKIs治疗，二代EGFR-TKIs治疗后的PFS分别为12个月和9个月。结合本研究中已知具体基因分型的32例双突变患者中有30例均复合罕见单突变基因，我们初步预测*EGFR*双突变患者经二代EGFR-TKIs治疗可以获得更好的预后。

本研究术后复发患者中，有3例仅接受EGFR-TKIs治疗，中位PFS为15个月，是否可以预测双突变术后复发患者应用EGFR-TKIs治疗预后较好。相关文献已经得出*EGFR*敏感单突变术后复发NSCLC患者应用EGFR-TKIs治疗能获得较好的预后。术后复发双突变患者是否与单突变患者拥有相同的治疗效果，仍需要进一步大样本研究验证。

综上所述，*EGFR*双突变患者的基因分型易合并罕见突变基因，以G719X/S768I突变型最常见。双突变患者临床特征与单突变患者之间无显著性差异。双突变患者接受一代EGFR-TKIs治疗的ORR、DCR、PFS均较单突变患者低，二代EGFR-TKIs可能更适应于*EGFR*双突变患者。由于*EGFR*双突变发生率很低，目前对于此类研究很少，本研究收集的样本量小，同时双突变又有许多不同的基因分型，在样本采集过程中无法获得所有的具体基因型，因此还需要进一步大量的研究来更加深入地了解*EGFR*双突变患者的临床特征及其对EGFR-TKIs治疗的反应从而指导临床实践。
